# Characterisation of the treatment provided for children with unilateral hearing loss

**DOI:** 10.3389/fped.2023.1197713

**Published:** 2023-07-25

**Authors:** Roshni Patel, Derek J. Hoare, Karen R. Willis, Shammas Tabraiz, Paul K. Bateman, Sally K. Thornton

**Affiliations:** ^1^ Hearing Sciences, Mental Health and Clinical Neurosciences, School of Medicine, The University of Nottingham, Nottingham, United Kingdom; ^2^ NIHR Nottingham Biomedical Research Centre, Nottingham University Hospitals NHS Trust, Nottingham, United Kingdom; ^3^ Children’s Audiology, Ropewalk House, Nottingham, United Kingdom

**Keywords:** unilateral hearing loss (UHL), children, hearing aids, contralateral routing of signal (CROS), bone-conduction device (BCD)

## Abstract

**Background:**

Children with permanent unilateral hearing loss (UHL) are an understudied population, with limited data to inform the guidelines on clinical management. There is a funding gap in healthcare provision for the children with UHL in the United Kingdom, where genetic screening, support services, and devices are not consistently provided or fully funded in all areas. They are a disparate population with regard to aetiology and their degree of hearing loss, and hence their device choice and use. Despite having one “good ear”, some children with UHL can have similar outcomes, socially, behaviourally, and academically, to children with bilateral hearing loss, highlighting the importance of understanding this population. In this longitudinal cohort study, we aimed to characterise the management of the children with UHL and the gaps in the support services that are provided for the children in Nottingham, United Kingdom.

**Methods:**

A cohort study was conducted collecting longitudinal data over 17 years (2002–2019) for 63 children with permanent congenital confirmed UHL in a large tertiary regional referral centre for hearing loss in Nottingham, United Kingdom. The cases of UHL include permanent congenital, conductive, mixed, or sensorineural hearing loss, and the degree of hearing loss ranges from mild to profound. The data were taken from their diagnostic auditory brainstem responses and their two most recent hearing assessments. Descriptors were recorded of the devices trialled and used and the diagnoses including aetiology of UHL, age of first fit, degree of hearing loss, when and which type of device was used, why a device was not used, the support services provided, concerns raised, and who raised them.

**Results:**

Most children (45/63; 71%) trialled a device, and the remaining 18 children had no device trial on record. Most children (20/45; 44%) trialled a bone-conduction device, followed by contralateral routing of signal aid (15/45; 33%) and conventional hearing aids (9/45; 20%). Most children (36/45; 80%) who had a device indicated that they wore their device “all day” or every day in school. Few children (8/45; 18%) reported that they wore their device rarely, and the reasons for this included bullying (3/8), feedback from the device (2/8), and discomfort from the device (2/8). Only one child reported that the device was not helping with their hearing. The age that the children were first fitted with their hearing device varied a median of 2.5 years for hearing aids and bone-conduction devices and 7 years for a contralateral routing of signal aid. The length of time that the children had the device also varied widely (median of 26 months, range 3–135 months); the children had their bone-conduction hearing aid for the longest period of time (median of 32.5 months). There was a significant trend where more recent device fittings were happening for children at a younger age. Fifty-one children were referred by the paediatric audiologist to a support service, 72.5% (37/51) were subsequently followed up by the referred service with no issue, whilst the remaining 27.5% (14/51) encountered an issue leading to an unsuccessful provision of support. Overall, most children (65%, 41/63) had no reported concerns, and 28.5% (18/63) of the children went on to have a documented concern at some point during their audiological care: five with hearing aid difficulties, five with speech issues, four with no improvement in hearing, three facing self-image or bullying issues, and one case of a child struggling to interact socially with friends. Three of these children had not trialled a device. We documented every concern reported from the parents, clinicians, teachers of the deaf, and from the children themselves. Where concerns were raised, more than half (58.6%, 10/18) were by schools and teachers, the remaining four concerns were raised by the family, and further four concerns were raised by the children themselves.

**Conclusion:**

To discover what management will most benefit which children with permanent UHL, we first must characterise their treatment, their concerns, and the support services available for them. Despite the children with UHL being a highly disparate population—in terms of their aetiology, their device use, the degree of hearing loss, and the age at which they trial a device—the majority report they use their device mostly in school. In lieu of available data and in consideration of the devices that are available to them, it could be useful to support families and clinicians in understanding the devices which are most used and where they are used. Considering the reasons for cessation of regular device use counselling and support services would be vital to support the children with UHL.

## Introduction

Within the United Kingdom (England, Northern Ireland, Wales, and Scotland), there is a current lack of national management guidelines for clinicians looking after children with unilateral hearing loss (UHL), and treatment is widely debated (1–3). Numerous organisations have established candidacy guidelines for paediatric amplification for the children with UHL (see Supplementary Material) and there is considerable variation in the guidelines with candidacy criteria ranging from 15 to 30 dB HL (4). Air or bone-conduction devices (BCDs) or cochlear implants (CIs) may be recommended at the upper limit of candidacy, for example, in single-sided deafness (SSD) where amplification is not possible, and for the children with UHL, many recommend a case-by-case decision approach (1, 46–48).

Uptake of devices for UHL can be variable with as few as 31% of children (9–18 years) with UHL using a hearing device (6). Low device uptake and current lack of guidelines most likely reflect the disparate nature of the degree of UHL, its aetiology, and the variety of treatment options available. The lack of guidelines poses a major problem for clinicians to advise the families of the children affected by UHL, since the impact of UHL can be highly variable across individual children as are their needs, desired outcomes, and personal preferences (7–9).

In 50% of the cases, the aetiology of UHL is unknown (10). There are several risk factors that have been associated with UHL (11) including premature birth, trauma, craniofacial anomalies, genetic causes, and bacterial or viral infections (12, 13). Children who have a family history of hearing loss are more likely to have bilateral hearing loss (BHL) whilst UHL is more likely to be present in those with craniofacial anomalies (8).

The listening difficulties that the children with UHL experience can mainly be explained by their loss of bilateral input. The range of loss of input can range from a child having no available hearing in their affected ear, often called SSD (weak or absent nerve for example), to very mild losses where they have some, albeit asymmetrical bilateral input. Their degree of hearing loss and aetiology will impact their device choice. The choice for a child to use a device and their device choice are also likely to be indicative of having an ear that works well and the ability of the child to use that monaural input to good effect. It can also reflect their listening environment, for example, if their device helps them in background noise or whether they are predominately in a quiet home and are able to position themselves for optimal listening. Counselling by a multidisciplinary team is advocated to avoid treatment bias for children with conductive UHL (14), and this can benefit children with either sensorineural UHL or aural atresia. However, it is critical to monitor speech and language development since they are at risk when listening with one ear and device trial should be implemented early (15). Furthermore, it is important to note that hearing is still limited with a BCD especially for children with SSD and/or in a noisy listening setting, possibly because the BCD does not restore the binaural hearing for children with conductive UHL (15, 16).

In a small study of children with Trisomy 13 and conductive UHL (mostly moderate hearing loss), the conservative approach of watchful waiting was often adopted, and when hearing aids were implemented, they were on the whole successful (14).

Where there is a lack of auditory input, this will impede binaural summation of loudness, the head shadow effect, and binaural release from masking which affects their ability to decode speech in noise and localise sound in space (15–19). In a recent study with children who have no access to sound in one ear (SSD) and who had a cochlear implant (CI), the implanted group exhibited an improved speech perception in noise and better sound localisation skills, compared with their non-implanted peers (20).

The morbidity of UHL can be similar to bilateral hearing loss (BHL), and there is a wealth of evidence that UHL can affect preverbal vocalisation, speech and language development, and cognition (8, 9, 21–25). The children with UHL can also struggle with listening fatigue particularly in noisy environments, and their degree of fatigue is very variable but can be very similar to the level of fatigue experience by children with BHL (26, 27).

There are a number of hearing devices that will not restore bilateral input but can be used to help the children with UHL—contralateral routing of signal (CROS) aids and bone-conduction devices help alleviate the head shadow effect as they re-route the sound via air or bone-conduction, respectively. BCDs and CROS-aids are commonly used for severe losses, fluctuating hearing loss, profound conductive losses or where the auditory nerve is absent or weak, whereas mild to moderate losses are often treated with conventional hearing aids.

The data from adult studies often imply that BCDs are better than CROS-aids but both devices impair speech perception when the noise emanates from the impaired ear side. Also, it has been shown that a BCD does not improve sound localisation abilities (30), but it is believed that they do improve speech perception when noise is on the side of the better ear (30–33).

A study assessing orienting head-movement responses in people with SSD investigated whether a BCD would jeopardise their directional hearing based on monaural spectral and/or level cues. They found that 5 out of 19 participants could localise certain sounds (broad-band and high-pass but not low-pass filtered noises) in the horizontal plane in the unaided condition and that a BCD did not deteriorate their localisation abilities (28).

The outcomes for the children with UHL can be as disparate as the aetiology and their type of hearing loss. For example, evidence of the impact of unilateral conductive hearing loss in children can be varied and sometimes mild, and there is limited evidence to indicate that hearing devices benefit every child in relation to their speech and language development. Aetiology is important since listeners with conductive UHL can activate the auditory pathways of their impaired ear through their own voice, and through bone and tissue conduction (29). If the UHL is conductive, BCDs can provide a form of binaural input where the cochlea is stimulated on the side of the loss as well as re-routing the sound to the better-hearing ear (when an inner ear hearing apparatus is functional). With the BCD, the cochleae can receive stimulation with negligible interaural attenuation so that a cross-hearing is experienced. The concomitant stimulation of the contralateral cochlea could impair the ability to process interaural differences in level and timing, which could limit improvements in the binaural hearing (30, 31). Prior studies indicate that BCDs provide speech recognition-related benefits; however, it is not agreed whether more accurate sound localisation occurs with BCDs (32, 33). With congenital conductive UHL, the data are contentious. There has been reported improvement in horizontal spatial hearing with a BCD for some listeners with conductive UHL despite the inherent problems of time delay and cross-hearing (32, 34), and one study shows that this can improve predominantly on the aided side with a BCD (35).

It was postulated that some listeners with congenital conductive UHL have adapted to their UHL and learned to rely on the spectral shape cues and monaural head shadow effect cues (36). In summary, how BCD stimulation affects spatial hearing abilities and the predictive factors that may affect the degree of the benefit provided by BCDs remains unknown.

Collation and comparison of data sets for paediatric UHL studies in the current literature can be difficult; a systematic review and consensus paper noted no firm evidence for the efficacy of current available devices nor evidence to inform decisions as to which devices are most suitable (5, 37). The limited data available suggest a trend towards improvement in speech perception with hearing devices, particularly with listening attention (35). Frequency modulation (FM) systems were shown to have the most benefit for speech recognition in noise, and studies evaluating CROS hearing aids demonstrated variable outcomes (38).

Several adult studies have demonstrated the long-term implications of uncorrected hearing loss; with an increased likelihood of experiencing social isolation and emotional distress as well as an increased risk of developing dementia in later life (39–41). Whilst the above implications are yet to be investigated in cases of early-onset UHL, other factors, for example, maternal education and earlier aiding, have been shown to ameliorate poorer outcomes (42). Watchful waiting should also be a possible option, particularly for milder hearing losses (14) as we have yet to show that treatment of UHL is associated with improved academic performance.

Future research may highlight the importance of early detection and appropriate treatment for some children with UHL.

To promote consistent and effective care for the children with UHL, it is important to both identify the current standard of care and support provided, as well as characterise the affected cohort of patients. It is also important to identify gaps in the funding and support for the children with UHL.

Moreover, an understanding of the respective thoughts of patients, parents, and clinicians on the care provided is essential to ensure a unified approach to UHL management.

In this current longitudinal cohort study, we describe all aspects, we can reliably ascertain from their data over 17 years in a large tertiary centre, on the management of children with confirmed permanent UHL. Consequently, there is a potential for further research into the future development of evidence-based management guidelines and promoting informed treatment decisions for clinicians, parents, and children.

The objectives of this study are the following:

1. Establish a database from the advent of NHSP (2002) to document the **demography and aetiology** of UHL in children in a tertiary referral centre.
2. Characterise **management of** UHL—e.g., type of device trialled, the age of the child when they first trial a device, when and how long they use their current device, and why they do not use their device.
3. Additional **support services** for the children with UHL and the documentation of any **concern** related to the impact of their hearing loss.

## Methods

The routinely recorded and collected data in this study formed part of a service evaluation 2002–2019 at Nottingham University Hospitals. The data was also used from the Nottingham research database (NEAT) under ethical approval to analyse routinely collected data (REC project ID: 292263), South Central Berkshire Research Ethics Committee.

### Inclusion and exclusion criteria

The criteria for inclusion included the patient referred on the NHSP and having a diagnosis of permanent UHL (hearing loss in one ear lasting for greater than 6 months). Children who did not speak English as a first language were also included.

The criteria for exclusion included children with acquired, fluctuating, or bilateral hearing losses as well as any child who did not have their new-born hearing screening (NHS) conducted in Nottingham.

Of the 89 cases identified as having UHL from the new-born hearing screening or referred from the UHL clinic, 26 (29.2%) cases were excluded as they were acquired or progressive, leaving a total of 63 cases of permanent congenital confirmed UHL. Of the 26 excluded, 13 cases were excluded for the reason that the hearing loss was progressive and developed to a bilateral hearing loss, 11 were acquired UHL, 1 case was both acquired and bilateral, and 1 case was excluded as the child did not have their new-born hearing screening in Nottingham.

### Classification of UHL

The cases of UHL include permanent congenital, conductive, mixed, or sensorineural hearing loss, and the degree of hearing loss range from mild to profound. The aetiology and severity of hearing loss could be identified in many cases, but we could not reliably determine the number of cases with conductive UHL since the tympanometry data were viewed to be not of high enough quality. The guidelines of the British Society of Audiology (BSA) were used to classify the severity of UHL in the poorer hearing ear (British Society of Audiology, 2018). Hearing loss in each ear was recorded and averaged over four frequencies (0.5, 1, 2, and 4 kHz). Occasionally, only the hearing level at two frequencies was recorded (most often 1 and 4 kHz) usually due to the attention or ability of the child to remain engaged in the testing (depending on their age or degree of development). Hearing loss in the affected ear was classified as Mild: between 20 and 40 dBHL; Moderate: 41–70 dBHL; Severe: 71–95 dBHL; or Profound: over 95 dBHL.

### Data extraction and analyses

The data were entered into an Excel spreadsheet from 89 patient paper notes (2002–2019) and electronic hospital databases (Practice Navigator, MedWay and NoTis) in a regional referral centre, Children's Audiology at Ropewalk House in Nottingham. The patient demographics included date of birth, sex, age at diagnosis of hearing loss, formal diagnosis of hearing loss, aetiology of UHL, and birth history. The results from the NHSP hearing test of the child and their diagnostic auditory brainstem response (ABR) as well as the two most recent hearing test results were recorded for frequencies 0.25, 0.5, 1, 2, 4, and 8 kHz, if present. When the data permitted, this was recorded for both ears. The age of the child at the hearing tests was recorded as was the method of testing—pure tone audiometry (PTA), visual reinforcement audiometry (VRA), and play audiometry. If the child had a hearing device, the device type, ear fitted, make and model, and date of first fit were recorded. For the patients who stopped using a device, the reasons for this were documented, and for those who had no device, the reasons why no trial was undertaken were noted. For device use, we catalogued when the device was worn and for how long, for example, none of the time, some of the time, at school, at home, or all the time, alongside any relevant quantification if present. The data-logging information from the hearing devices was not available. The support service data was also documented and included the teachers of the deaf (ToD), speech and language therapy (SLT), missed appointments, and comments made by the relevant parties relating to the management of the child and their respective outcomes.

We documented every concern reported from parents, clinicians, ToD, or the children themselves. Where data were of poor quality, verbatim quotes were lifted from patient notes.

### Missing data

The cases of UHL may have been missed if the NHSP of the child took place out of the area, or if the child was not referred for diagnostic follow-up following a UHL found on the new-born hearing screening. The data were not available if the parents did not engage with audiology services after the initial appointment and diagnosis of UHL for their child.

The onset of hearing loss was categorised as congenital or acquired. Those with an acquired hearing loss were defined as developing hearing loss after having passed their new-born hearing screening. Congenital hearing loss was defined as children who were referred on the NHS as a neonate and then went on to have a confirmed permanent hearing loss by ABR and then later VRA or play audiometry (depending on the age or developmental ability of the child at the time of testing). The average of the hearing loss of the child (across frequencies, recorded in dB SPL) was recorded for both ears, and the data were used from the most recent hearing test (most often PTA). Where this information was not available, then the data from the second latest hearing test were used. In a number of cases, the most recent hearing tests only documented the data from the better-hearing ear (usually when the poorer ear was described as congenitally “dead”), in which cases the *current* severity of hearing loss remained unknown and these cases are documented as severe-profound*. These are listed separately in Tables 2, 3.

The software used included Microsoft Excel (Internet) version 2302 to record the data. The Kruskal–Wallis (K–W) non-parametric ANOVA for multiple comparisons and Pearson correlation (*r*) were performed using SPSS version 28.0. The GraphPad Prism version 8.0 was used to graph the data.

## Results

Of the total 89 cases of children with hearing loss identified between February 2002 and March 2019 analysed in this study, 77 children had permanent congenital hearing loss, 12 had an acquired hearing loss, and 63 children were identified with permanent congenital UHL.

1. Demography and aetiology: Of the 63 children with permanent congenital UHL, 50.7% (32/63) were male and 49.2% (31/63) female. Birth complications were recorded in 34.9% (22/63) of the cases where the most common were C-sections (36.3%; 8/22) and NICU involvement 31.8% (7/22). The aetiology of the hearing loss and the number of birth complications reported are shown in Table 1.

**Table 1:** Aetiology of UHL: craniofacial abnormalities including atresia, microtia, maldevelopment of labyrinthine structures.

Aetiology	Frequency (*n* = 32)
Craniofacial abnormality	18
Malformation or absence of auditory nerve	7
Post-infection	4
Genetic	4
Birth complications	22
Caesarean section	8
NICU admission	7
Jaundice	3
Prematurity	3
Breech	2
Meningitis	1

NICU, neonatal intensive care unit.

Birth complications were present in 22 cases of the children with UHL. More than one birth complication may be documented for each child.

The known aetiologies for UHL in the children were documented in 50.7% (32/63) of the cases. The aetiologies were broadly classed into categories and described in the notes as follows: craniofacial—structural malformation 56.3% (18/32) including atresia, microtia, and poor development of labyrinthine structures; malformation or absence of auditory nerve 21.9% (7/32); post-infection 12.5% (4/32); and genetic 9.38% (4/32).

The genetic testing revealed four pathogenic variants, and two had variants of unknown significance. The genetics data have previously been cited in a recent study (44).

With regard to the hearing severity of the poorer ear of the child, 1.6% (1/63) was mild, 31.7% (20/63) moderate, 15.9% (10/63) severe, and 15.9% (10/63) profound. In the remaining 34.9% (22/63), the degree of severity of their hearing loss was not documented in their most recent two hearing tests. In these 22 cases, the poorer ear remained untested for a prolonged period, sometimes since the diagnostic ABR as in the majority of the cases, it was recognised that the ear was effectively “dead” due to a number of reasons. We do not have the current degree of hearing loss recorded for this group, so we have labelled this group separately in Tables 2, 3 as severe-profound*.

**Table 2:** A hearing loss in relation to device trial.

	Mild	Moderate	Severe	Profound	Severe-profound*
(A) Trial/severity					
Device trial, *n* = 45	0	16	2	8	19
No trial, *n* = 18	1	4	8	2	3
(B) Hearing loss in relation to device type, *n* = 45					
HA, *n* = 9	0	9	0	0	0
BCD, *n* = 20	0	5	1	3	11
CROS, *n* = 16	0	2	1	5	8

The severity of hearing loss is for the poorer ear only and is categorised according to the BSA guidelines.

* Severe-profound group: the poorer ears for children in this group have not currently been tested (see Methods).

**Table 3:** The severity of hearing loss is for the poorer ear only and is categorised according to BSA guidelines. *Severe-Profound group: the poorer ears for children in this group have not currently been tested. The most recent hearing tests only documented data from the better hearing ear (usually when the poorer ear was described as congenitally ‘dead’), in which cases the current severity of hearing loss remained unknown and these cases are documented as severe-profound* and listed separately in the table.

Frequency of use	Not at all/rarely, *n* = 8 (%)	All day, *n* = 36 (%)
Hearing loss		
Mild	0 (0)	0 (0)
Moderate	2 (25)	13 (36)
Severe	1 (12.5)	1 (3)
Profound	1 (12.5)	7 (19)
Severe-profound*	4 (50)	15 (42)
Device used		
BCD	3 (37.5)	16 (44)
HA	3 (37.5)	6 (8)
CROS	2 (25)	14 (39)

Degree of hearing loss is the hearing loss in their poor ear and is categorised according to the BSA guidelines (see Methods). “All day” includes when the device was reported to be worn all day in school.

Most children with UHL who trialled a device had either a moderate hearing loss 36% (16/45) or were in the severe-profound category of hearing loss (19/45; 42%). Most of those not trialling a device had severe hearing losses 44.4% (8/18) (Table 2A). There were five children who would have had an aidable mild (*n* = 1) or moderate (*n* = 4) loss in their poorer ear but who never trialled a device. Only the children who owned and reported that they wore a hearing aid (HA) had a moderate degree of hearing loss (*n* = 9); however, there were children with moderate losses who also used a BCD (*n* = 5) or a CROS-aid (*n* = 2) (Table 2B). Of the two children with severe losses, one used a CROS-aid and the other used a BCD. Three children with profound hearing losses used a BCD, whereas five children used a CROS-aid. More than half of the children who had severe-profound losses* were currently using a BCD (*n* = 11), and eight children used a CROS-aid (Table 2B).

### Management of UHL

**Figure 1:**
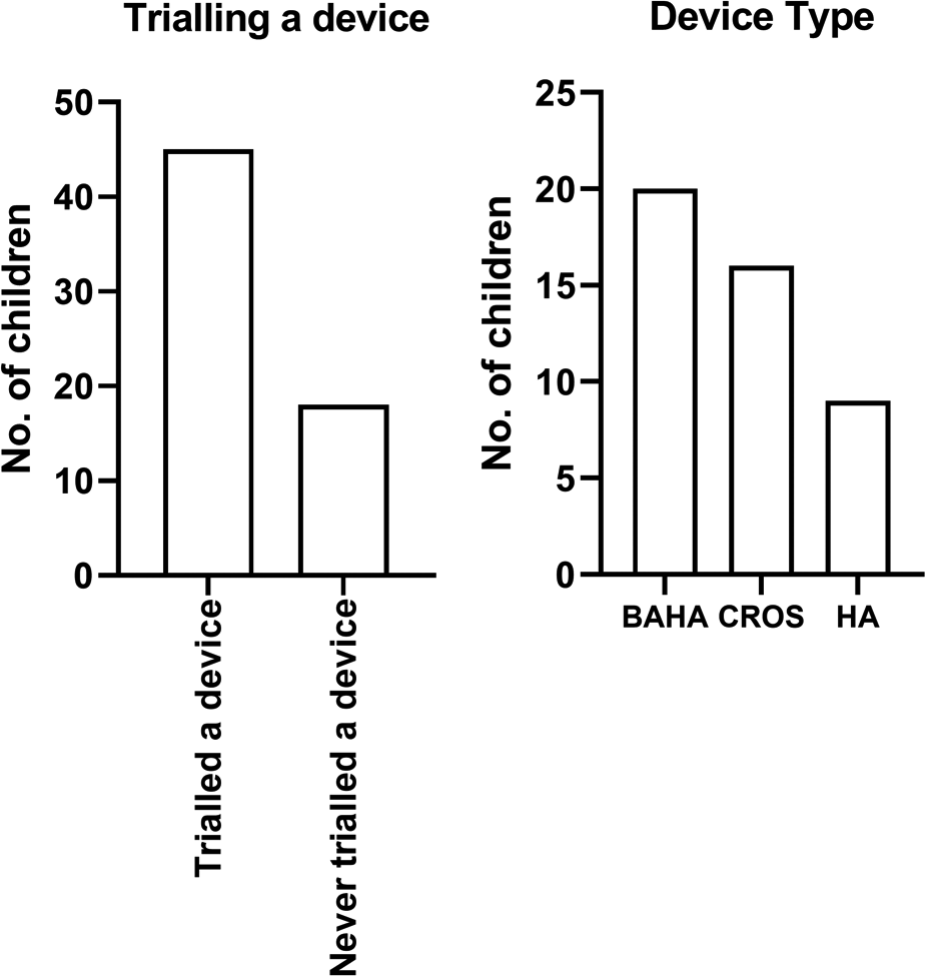
The number of children who trial a device and the device type trialled.

Forty-five children (45/63; 71%) with UHL trialled a device with the remaining 18 (18/63; 28.5%) children having had no device trial (Figure 1). Twenty (20/45; 44%) children trialled a BCD, 16 (16/45, 36%) a CROS-aid, and 9 (9/45, 20%) conventional hearing aids.

**Figure 2:**
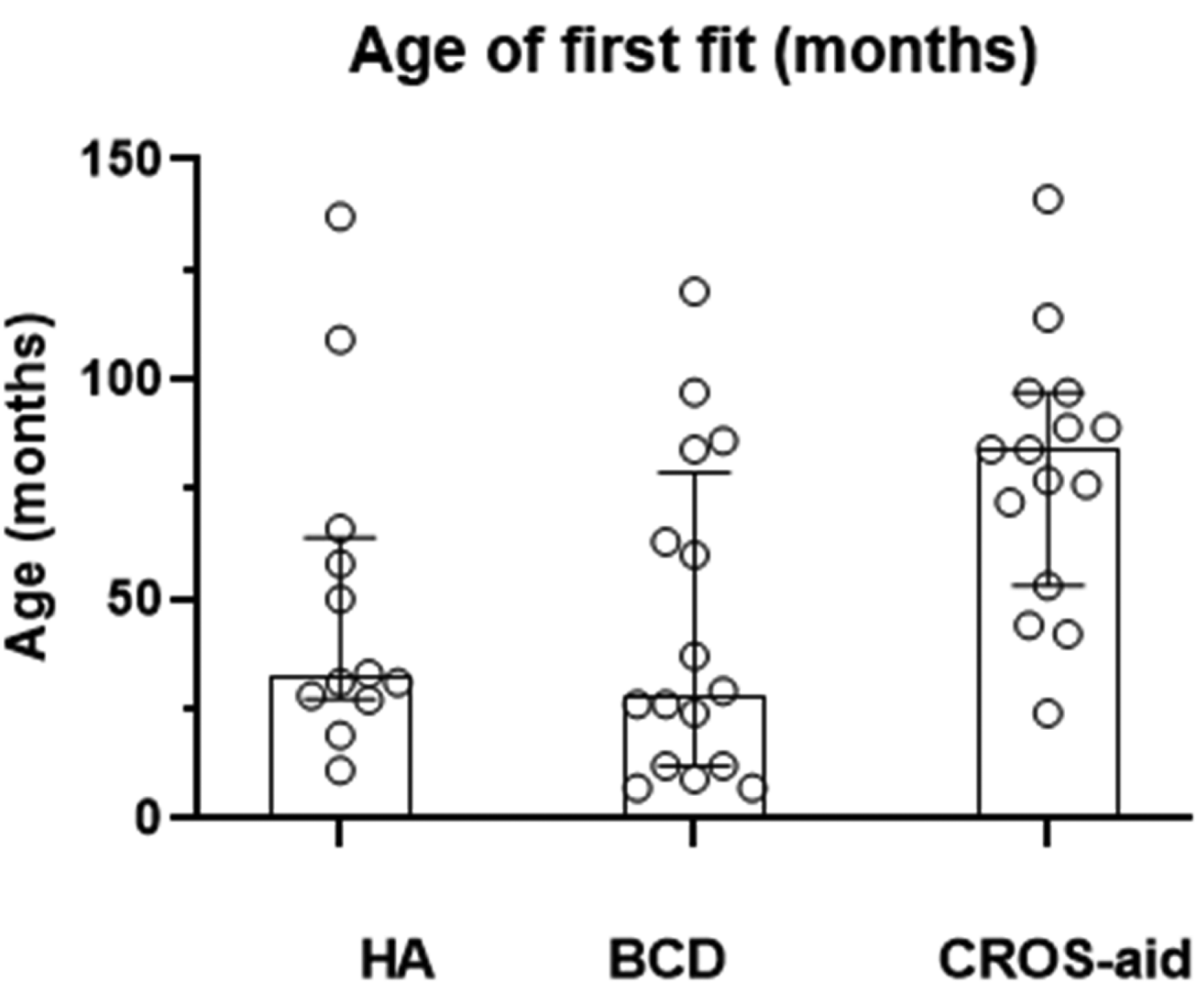
When a child is first fitted with their hearing device. The age of first fitting of the device (in months) for each child is represented by an open symbol for children who have a HA, hearing aid; a BCD, bone-conduction device; or a CROS-aid, contralateral routing of signal aid.

The range of ages at first device fit was wide with a mean age of 56 months or 4.7 years (*n* = 41). The ages of the child when they are first fit with their first device are plotted individually in Figure 2, and the overlain boxplots illustrate the median and interquartile ranges (IQR) for age at first fit by device type. A significant difference between the groups was found (K–W ANOVA, *K* = 8.4; *P* = 0.015). The medians and minimum–maximum ranges (in months) for age of first fit within device types were the following: HA (median, 32 months; 11–137 months), BCD (median, 28 months; 7–120 months), and CROS-aid (median, 84 months; 24–141 months).

#### When a device is worn and who leads the device choice

**Figure 3:**
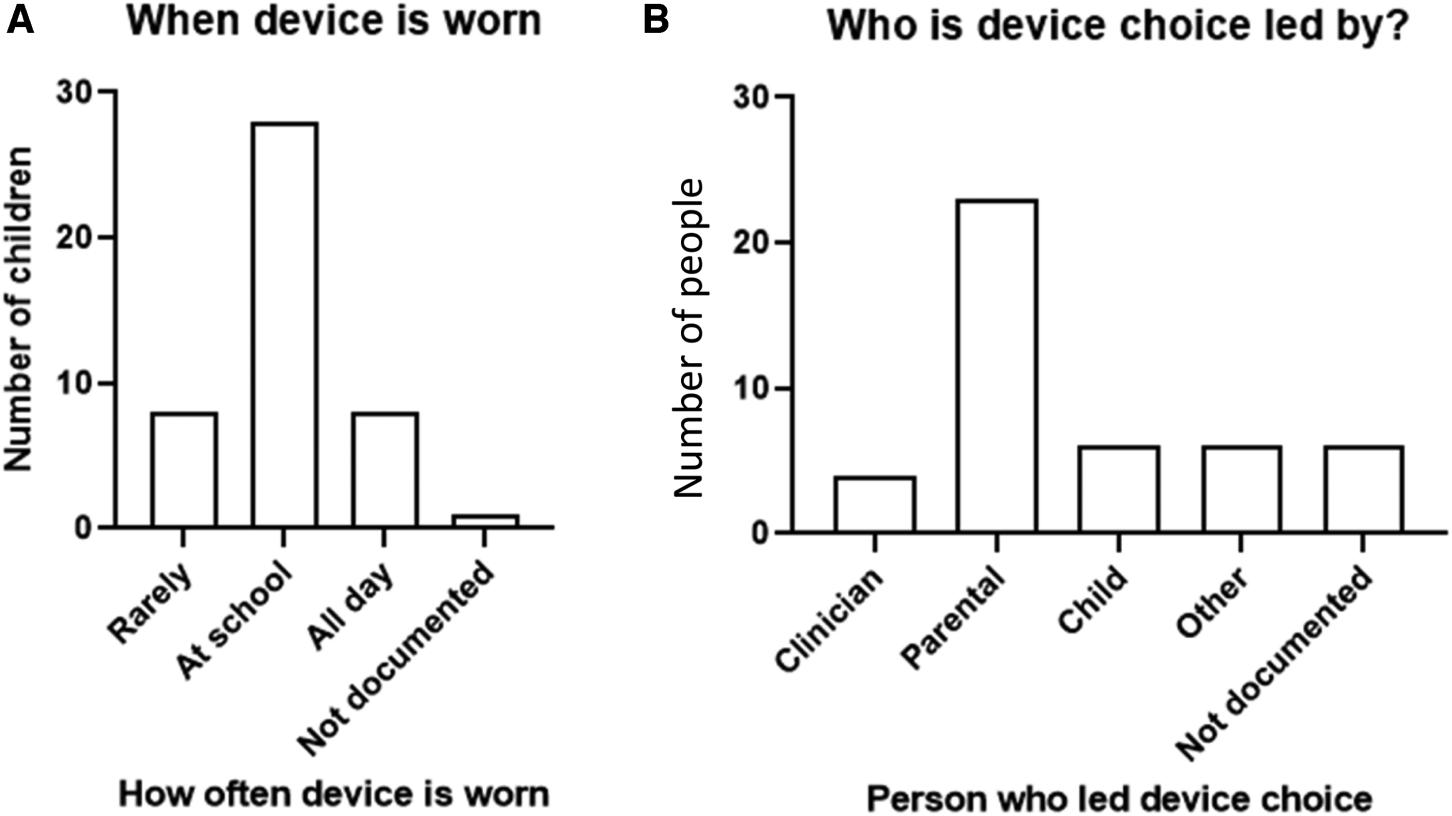
(**A**) When a child with UHL wears their device; (**B**) device trial lead, *n* = 63.

A device was reported to be predominately worn “at school” or “all day” (Figure 3A). A parent-led decision for the device trial was documented in half, 51% (23/45) of all the cases, followed by child-led decision 13.3% (6/45) and clinician-led decision 8.9% (4/45). In six cases (13.3%, 6/45), “other” represents cases where the overall lead for the decision in these cases was unclear and likely to be a joint decision between the clinician, parent, and child (Figure 3B). In six cases (13.3%, 6/45), no reason was documented for device trial.

In the 36 children with UHL who wore their device all day, 13 had moderate hearing loss, 1 had severe, 7 had profound, and 15 had an unknown degree of hearing loss, likely to be severe-profound* (Table 3). BCDs were most used by children who wore their device all day, including at school (16/36, 44%), followed by CROS-aids (14/36, 39%). Six (6/36, 8%) children wore their hearing aids all day, including at school.

**Figure 4:**
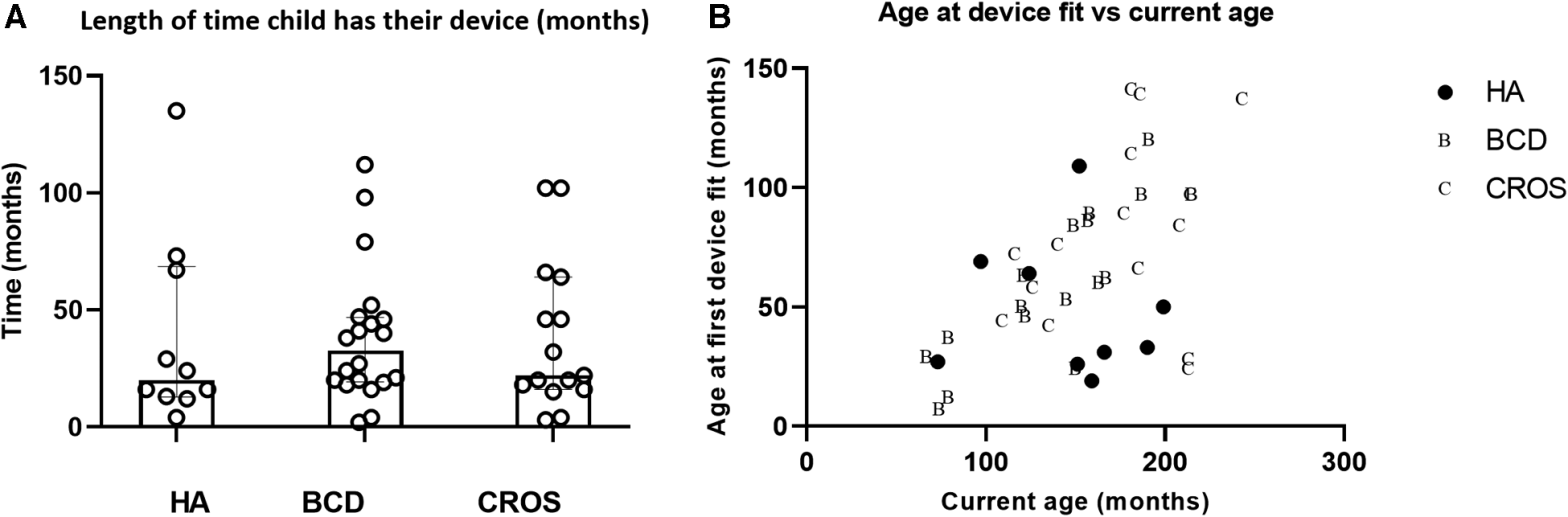
(**A**) Length of time was approximated to the child's last hearing test minus the date of their first trial of their device. Medians and interquartile ranges plotted in (**B**). In (**B**) age when the child was first fitted with their device (in months) is plotted against their current age in months. Filled symbols represent children with a hearing aid, letter B for children with a BCD and C for children fitted with a CROS aid. HA, hearing aid; BCD, bone-conduction device; CROS-aid, contralateral routing of signal aid.

The children kept their BCDs for longer periods of time (median 32.5 months), but this was not statistically significantly longer than for HAs (median 24 months) or length of time they kept their CROS-aids (median 22 months) (*n* = 44; Kruskal–Wallis ANOVA *P* = 0.91). Across device types, the length of time a device is used is widely varying (Figure 4A); however, these data give no indication of whether the device was worn or not. Nevertheless, the child would return to Paediatric Audiology to have their hearing device serviced and updated, and if it was stated that the device was not being worn, it would be returned to the service. Unsurprisingly, there is a positive correlation between the age of first device fit and the current age of the child (*r* = 0.52; *P* = 0.0004) (Figure 4B)—useful to note as it shows that there is a trend where children who are born more recently are more likely to be fitted at a younger age. Also, younger children were more likely to be fitted with a BCD or a HA than a CROS-aid.

### Children who have a device but rarely wear their device

There were eight children who have had device trials and currently wear their device but only infrequently. For the three children, it was documented that infrequent wearing was because they experienced bullying wearing their hearing devices (and/or appearance of their ear), two children experienced feedback from their device, two found them uncomfortable to wear (one also found the sound robotic and stopped wearing because of appearance), and one found their HA not helpful with their hearing.

#### Not trialling a device

In terms of their aetiology, the majority who have not trialled a device in this group have external or inner ear abnormalities (*n* = 5), two have auditory neuropathy spectrum disorder (ANSD), three have sensorineural hearing loss, one had CMV, and one had a skull fracture as an infant. In the six cases, the aetiology was unknown or not documented.

Of the 18 children who did not trial a device, it was discussed between the child and the parent in 12 cases. In 50% (9/18) of the cases, the parent decided for the child not to trial a device, and it was an ENT clinician-led decision (mild UHL) in one case (5.6%, 1/18). A further two cases were joint clinician and parent-led decisions, and six cases had no documented reason. However, despite there being no documented reason for “no trial of a device,” it was reported in three cases of the child doing well, having a good understanding of speech and no developmental concerns. One child was lost to follow-up.

### Follow-up during audiological care

#### Support services

The percentage of children who had a documented referral to a support service was 81.6% (51/63); the remainder had no documented support referral.

Of the 51 children who were referred, 72.5% (37/51) received their intended support with no issue, whilst the remaining 27.5% (14/51) encountered an issue leading to an unsuccessful provision of support. The documented obstructions to this support were 86% (12/14) due to the child not meeting referral criteria and 14% (2/14) due to parental refusal of support. Overall, 93 support referrals were made: 74% (69/93) for a teacher of the deaf and 20.4% (19/93) for speech and language therapy. A further 5.38% (5/93) to “other” services. Within these 51 children with referrals, most, 37/51 (72.5%), had combined referrals to several support services and 14/51 (27.5%) had a single referral to a specific service.

### Documented concerns surrounding UHL

A total of 65% (41/63) of the children with UHL had no reported concerns, and 28.5% (18/63) of the children had a documented concern: 5 children reported difficulties with their hearing devices, 5 had problems with their speech, 4 had no improvement in their hearing, 3 faced self-image or bullying issues, and 1 case of a child struggling to interact socially with friends. In three cases, the reason for the concern was not documented.

Where a concern was raised during audiological care, the hearing level severity included 0% (0/18) mild, 17% (3/18) moderate, 17% (3/18) severe, 17% (3/18) profound, and 50% (9/18) of the cases had an unknown degree of hearing loss—likely to be severe-profound*. Furthermore, in the 18 children where a concern was reported most trialled a device, 8 children had trialled a BCD, 4 an HA, 3 a CROS-aid, and 3 children had no device trial on record. Within these 3 cases with no documented device trial, 2 cases received documented support service input. Only in one case did a child with raised concerns has no device trial and no support.

Half, 56% (10/18), of the concerns were raised by schools and teachers, 22% (4/18) of the concerns were raised by the family, and a further 22% (4/18) of the concerns were raised by the children themselves.

## Discussion

A number of organisations have established candidacy guidelines for paediatric amplification, but the guidelines for the children with UHL remain more ambiguous, and recommendations vary (4). Currently, there are no national clinical practice hearing guidelines surrounding the treatment and management of the children with UHL in the United Kingdom. Most guidelines specify criteria for amplification as audiometric threshold levels and considering the disparate nature of the condition, the treatments, and its aetiology, and it has been postulated that individually treating each child is optimal (45).

Most children with permanent congenital UHL in our cohort wore a device, most often a BCD or a CROS-aid; they wore it at school or all day indicating that, at least for school age children, a trial of a device should be prioritised. In our cohort, it was the parents who usually made the decision for their child to trial a device. Many children with UHL are at high risk of certain adverse developmental outcomes (9, 23), thus funding should be made available for all the children with UHL who need a referral to the support services that they require and for their preferred device.

The prevalence of hearing loss in one ear is estimated at 0.3–1 per 1,000 births (46); our estimation for this cohort is 0.4 per 1,000 births. The prevalence may be higher as UHL cases (particularly mild UHL) go undetected or under-reported; NHSP guidance in the United Kingdom does not aim to identify milder hearing losses, but as in the United States and Canada, milder losses may be identified as a by-product of the screening procedures. Furthermore, the parents who did not engage with the audiological service following identification of UHL on the NHSP are not included in this study.

To improve the current standard of care, it is first important to identify the cohort of patients affected and to understand the treatment provided. This study provides much needed information about current practice and the reasons behind the interventions and choices made by the care team of the children and their families in this central tertiary regional referral centre over a 17-year period.

### Demography and aetiology

Similar to previous studies, we found that the aetiology of UHL was documented in half of the cases and over half of these were structure abnormalities related to the ear, 22% had absent or malformed auditory nerve, 12% post-infection, and 9% genetic causes. The aetiology and degree of hearing loss are very important to ascertain as they can impact device choice for the child with UHL. It is established in the literature that congenital anomalies, for example, craniofacial abnormalities and ear malformations, are common in this population (12, 47), and the prevalence of malformations in the inner ear and/or internal auditory canal are higher in infants with congenital UHL than in infants with bilateral hearing loss (48), with two-thirds of the children with UHL having inner ear and/or internal auditory canal malformations (49). The structural abnormalities of the inner ear structures can mean that the conductive losses are sufficient to warrant the fitting of CROS-aids or BCDs. Aetiology is an important consideration since more than half of the children in this study who had never trialled a device had ANSD or abnormalities of the external or inner ear. Furthermore, where a concern was raised during audiological care, 50% of the cases had an unknown degree of hearing loss—likely to be severe-profound*—and these are the cases where the hearing was non-functioning from birth.

Previous studies have revealed that the percentage of UHL associated with a family history is approximately 3.7%–13% (48, 50, 51), which is similar to the number of UHL cases with genetic variants in this study (9%). Currently, genetic screening is not recommended or funded for all infants diagnosed with UHL in the United Kingdom, only for those where a syndrome is suspected. There is little to no research on the specific genetic variants associated with UHL. Three of the four patients with a genetic variant also had a diagnosed syndrome, suggesting that the syndromes may be linked to specific genetic variants (52). UHL and a NICU admission can be a red flag for additional congenital anomalies and developmental delay (44).

### Characterisation of the management of treatment for UHL

In this study, most (71%) children with UHL trialled a device, which in some cases is higher than pre-existing literature. In Purcell's study of 50 children, aged 5–19 years, a similar number to our study (*n* = 34 children; 68%) had trialled a hearing device. Fewer, however, (*n* = 20, 40%) continued to use their device. In a study of 31 children with congenital, acquired, or unknown onset UHL (age range: 1–10 years), it was reported that 81% of the children with moderately severe or better UHL accepted the use of a hearing aid. However, when the UHL was severe or profound, the parents reported very poor or no use of the hearing aid (53). One reason could be that the data we report are more recent compared with the previous studies. Also, we include all types of devices and a wider age range (0–18 years), and we did not include the children whose parents did not engage with audiological services after the initial diagnosis of UHL.

Appachi et al. evaluated auditory outcomes from various modalities of hearing rehabilitation, including FM systems, hearing aids, CROS-aids, and BCDs, where the use of FM system was beneficial for speech recognition in noise, and hearing aid use showed a trend of improvement in speech perception. CROS-aid use was associated with mixed auditory outcomes. BCD use was associated with consistent gain in speech reception thresholds (SRTs) and speech discrimination, and an improved hearing in noise, but findings for sound localisation were inconsistent. Similarly, Liu et al. systematically reviewed the role of BCDs and found consistent gain in SRT and speech discrimination, but inconsistent results with sound localisation. The measurement of the quality of life showed a high rate of usage and benefit in the learning domain.

The age of the first device trial was disparate and depended on the device type. It also depended on the age of the child where the children who have been seen more recently (younger) were fitted earlier, most likely due to change in management over time. The average age of fitting of a device was around school age (4.7 years); BCD and HA can be fitted earlier and were fitted around preschool age. This age of fit was later in comparison with other studies for children with BHL, for example, Walker et al. reported an average age of fitting at 10.99 months (range: 5 months to 7 years, 3 months) within a cohort of 211 children identified with BHL (54). However, it should be noted that the ages of their participants were skewed to the younger age group than that of the current study. There is a disagreement in the literature concerning the best age to fit a child who has UHL, and a case-by-case approach is optimal (1, 46–48).

One interpretation of the wide range of the ages of first fit in this study indicates that the children with UHL are followed up, and when they have trouble, they have no problem returning to audiology services and requesting a trial even into their teenage years.

Current NICE guidelines indicate those babies who are confirmed deaf by the NHSP should receive a hearing aid within 2 months. Meanwhile, the British Academy of Audiology provide no fixed time over which amplifications should be provided, stating “amplification be provided in accordance with family centred care guidelines” (45). Whilst there is little evidence to suggest early aiding is beneficial to *all* the children with UHL, there is some evidence to suggest that wearing a hearing device can improve the quality of life, especially in those suffering with speech and language or academic and behavioural issues. A few studies have shown that early intervention may be crucial particularly for speech reception thresholds and speech discrimination, especially in noisy environments (33, 55, 56); however, the data measuring improvement in sound localisation with aids are inconsistent. A study that examined the quality of life measures reported a high usage rate of BCDs among children (33) whereas another study found low usage (1.3 h/day) but with an improvement in CHILD scores and speech in noise testing following amplification with a BCD (52).

Other studies have found that hearing devices may not be beneficial for younger children with UHL and do not improve speech recognition (33). Such contrasting evidence is also mirrored in our anecdotal data, with one parent stating that their child is “happier and much more responsive” with hearing aid use (case 59) and another reporting they are “unsure of the hearing aid and are unable to notice a difference” (case 89). As such, more research is needed for the best age to trial a device for the individual child with UHL.

The uncertainty about age at which to fit a hearing device is complex and is dependent not only on the importance of stimulating the binaural neural pathway during development but also the degree and aetiology of hearing loss. For some children, bilateral stimulation can only be achieved with a CI (not yet commissioned in the United Kingdom for UHL/SSD, and no CI use is reported in this study). CROS-aids have a very different role, and evidence for the benefit of the early fitting of CROS-aids for children is not available (in the United Kingdom, they are not fit before 6–7 years of age). CROS-aids do not stimulate the unaided ear and only provide access to the acoustically blind area, so there is no binaural access to sound. It is thought that CIs for severe-profound losses and hearing aids for mild–moderate losses are vital to stimulate the auditory pathway during the crucial periods of neural development for the acquisition of speech and language (4, 56, 57).

With regard to the devices used by the children in this study, nearly half wore BCDs, a third wore CROS-aids, and a fifth wore conventional hearing aids often reflecting the age of the child, aetiology, and type of hearing loss experienced. No children in this cohort had a cochlear implant despite their associated benefits seen in children and adults with severe-profound UHL (60, 61). This is likely due to current lack of funding for cochlear implantation for UHL in the United Kingdom and may also reflect limited evidence. For the children who trialled devices, very few did not wear their device, and of these cases, the degree of severity was spread across moderate, severe, and profound groups with no clear majority, similar to a prior study (24). Most children with moderate losses used a hearing aid. There were no children with mild, severe, or profound losses currently using a hearing aid. Unsurprisingly, all children with severe, profound, or severe-profound* losses currently used a BCD or a CROS-aid. The children who have profound losses were more likely to have a CROS-aid (63% CROS; 37% BCD), and the children with severe-profound* losses were more likely to have a BCD (58% BCD; 42% CROS). We do not know why most children who did not trial a device in our cohort had a severe level of hearing loss. One possibility is that mild and moderate losses are easily treated with hearing aids and profound losses with BCD or CROS-aid whereas severe losses fall between the two and thus there could be indecision about the best device to trial.

Most children who wore a device reported that they wore it all day or at school. A parent-led decision for device trial was most often documented (51%), followed by child-led decision (13%) and clinician-led decision (9%). For the children who did not trial a device, half were documented as a parent-led decision, very few were clinician- or child-led decisions. With regard to “decision to trial a device,” specific decision making and counselling were often poorly documented and unclear. It is likely that the decision to trial a device is complex and child-specific; further investigation would be useful into how we could better understand both parental and child concerns. This may be particularly important in cases where device was not trialled or parents were not engaged with audiological services. Funding is also a point of concern as with limited resources, a CROS-aid or hearing aid is much more economical than a BCD. There are no official recommendations in the United Kingdom for CROS-aids, but there is a practical consideration when fitting them to young children. CROS-aids are best fitted on open fit slim tubes so that the sound to the better ear is not attenuated. Young children do not have the appropriate size of ear canal to enable fitting of open slim tubes, and hearing aid manufacturers do not make domes/slim tubes small enough for the ear canals of children. Furthermore, due to the small removable components (e.g., domes), there are choking hazards for small children. If CROS-aids were fit to a small child, an occluding ear mould would be required to aid retention (rather than the ideal open fit slim tube), and the occlusion effect would need to be overcome in the hearing aid programming. For these reasons, CROS-aids are not fit for children under 6 years old in Nottingham, United Kingdom. The children with severe and profound losses are not usually fitted with conventional hearing aids because of interaural attenuation due to the high levels of gain required, resulting in a cross-hearing into the better ear and therefore likely distortion of the sound perceived in the normal hearing ear.

Our data illustrate the importance of BCD and CROS-aids and are in contrast to an early study where 27 children with UHL who were fitted with an HA but usually did not wear it, 26% reported wearing it all of the time, 4% reported wearing it only in school, and 50% reported never wearing it (62).

The data that predate commissioning of the BCD use for UHL found that hearing aid use was high (*n* = 31; 81%) *but* only for users with moderately severe or better levels of hearing loss, whereas children with severe or profound UHL had poor or no use of hearing aids (53).

This is likely because those for whom a BCD or CROS-aid would be particularly beneficial did not have access to those devices at that time.

Whilst it can be beneficial for children to be wearing their devices throughout the day, self-reported use of hearing aid frequency can be inaccurate. It has been shown that although the estimates and data-logging of the parents were significantly correlated, the results indicated that the parents overestimate the amount of time their children wear their hearing aids by about 2.5 h (47). In a recent study of babies and carers, no correlation was found between hours of daily hearing aid use and self-reported hearing aid management skills or factors having a negative impact on hearing aid use (63).

It is possible in this current study that both child and parental reports of device use could be misleading. However, the children with UHL in this study were overall older than the children reported in prior studies, and it has been shown that longer hearing aid use relates to older age, poorer hearing, and higher maternal education (42, 54). Future recording of data-logging and environmental assessments from technology and hearing devices would enable greater insight into the device use for the children with UHL.

Where it was reported that the children only rarely wore their devices, in this current study, the predominant reasons were because of bullying at school, feedback from the device, and discomfort. A prior study of 15 children with UHL found that the most common reason for the cessation of device use was discomfort, followed by lack of benefit (64). The children with UHL may also be particularly vulnerable if they have had surgery for craniofacial anomalies. In a recent study, hearing impairment among adolescents was associated with increased reported rates of bullying victimisation: 34% of children with hearing loss were bullied, and children who do not use hearing aids had even greater odds of being bullied (OR = 2.40, 95% confidence interval: 1.18–4.86, *P* = 0.015) (65). It is difficult to know if the non-hearing aid users were not using their devices because of the bullying or if their speech and other developmental problems were the cause of them being targeted. Further research is needed to investigate how anti-bully interventions can support this vulnerable group. A 2019 meta-analysis and systematic review found that school-based anti-bullying programmes significantly reduced bullying perpetration and victimisation; this could be vital for all children with hearing loss (66).

### Follow-up during audiological care, support services

More than half of all children in our cohort had a referral to a support service; this is in keeping with most of the current literature. A previous study indicated that 39% of the children with permanent UHL received speech therapy, 54% had received an individualised education programme (IEP), and 36% had received additional educational assistance (*n* = 46, ages 6–12 years) (67). In a later study, the need for further academic support was identified in this population—more children with UHL received IEPs (45%) and speech therapy (41%) than the children with normal hearing (5% for both IEPs and speech therapy) (68). Three quarters of these children received their intended support with no issue, whilst the remaining quarter of the children with UHL (*n* = 14) encountered an issue leading to an unsuccessful provision of support. The majority (86%) of documented obstructions to this support were due to the child not meeting referral criteria, defined by local support teams, only two cases were due to parental refusal of support. The criteria for referral are defined by the support service rather than audiologists—this is an area of inconsistency that needs further evaluation.

### Documented concerns surrounding the UHL of the child

A fifth of the children with UHL went on to have documented concerns; these included speech and language problems, self-image or bullying issues, hearing aid difficulties, and/or poor social interactions with friends. The majority (40/51) of the children with UHL that had a referral to a support service had trialled a hearing device. Whilst the struggles faced by each child differ, thematic analysis (69), using a focus group comprising of a mix of children with permanent hearing losses, their parents, and audiologists, suggests that there are six main domains in which hearing loss can affect children. These include behaviour, feelings, environments, social/activities, family, and hearing equipment. The anecdotal data from our own study are in line with these findings highlighting the universal impact of hearing loss on children as well as the consequent importance of ensuring their adequate support. Within adults with UHL, core rehabilitation outcomes include the following: (1) spatial orientation, (2) group conversations in noisy social situations, and (3) impact on social situations (70). Consequently, it would be beneficial to identify the core outcomes for the children with UHL; in doing so, management plans can be better tailored and the outcomes of the child more easily monitored and assessed.

### Guidelines and funding for the children with UHL

Given the lack of UK National Health Service and NICE guidelines for the management of paediatric UHL, audiologists often use SLT or a diagnosis of developmental impairment to guide them on their treatment plan, and to suggest to the parents that their child should trial a device. Unfortunately, by the time the child requires SLT, this may be too late, especially when early device trials may impact speech and language acquisition, as has been noted by the LOCHI study (42). Most device trials in our cohort were parent-led, and without counselling, it may be the case that the parents are more willing to trial a device if they see the adverse developmental effects of UHL. Deciphering speech in noise is particularly tricky for the children with UHL, and therefore in a noisy home or at preschool, it would be strategic for the children with UHL to use a hearing device. Also, certainly in school/preschool, a sound-field system would benefit all children regardless of device use. Currently, there is consistent funding neither in the National Health Service nor in education authorities for FM/remote microphone systems, sound-field systems, and support, including SLT for the children with UHL.

More than half of the concerns about a child with UHL were raised by teachers, and a fifth were raised by the family, and a further fifth were raised by the children themselves. This is likely due to teachers spending most of the day with the child in an environment wherein auditory cues are paramount, and they may also be highlighting the developmental delay of the child and paucity of support in school. The parents/families/clinicians may also seek their advice from specialist interest groups, social media, and charitable web sources such as the National Deaf Children's Society.

The recognition of hearing impairments within schools and the consequent supporting facilities they can provide is also likely to affect the response of the child. Prior to the NHSP, the school hearing screening programme (SHSP) was used throughout the United Kingdom to recognise the children with undiagnosed hearing impairments (42). School age screening continues in many, but not all, parts of the country. Within the United Kingdom, Fortnum et al. have found the diagnostic accuracy of school hearing tests not to be cost effective (71) and that the distinct lack of quality data numbers is one of the reasons that funding for the SHSP has since ceased. Unfortunately, the late identification of hearing loss is likely to be of detriment to the children with acquired forms of hearing loss (72–74). However, screening and early diagnosis is only the start. The availability of high-quality early years support for hearing loss remains a major barrier to the progress of the children. Failure to provide this high-quality support means that the potential benefits of new-born hearing screening are not being realised consistently across the United Kingdom. Almost a third of families did not feel they got the support they needed to make sure their child made good progress and developed well after diagnosis through new-born hearing screening (75). This is perhaps why, in comparison, the siblings of the children with UHL have been shown to perform better in a number of domains (behaviourally, socially, and academically). One-fifth of the children with UHL were diagnosed with developmental delay (76).

### Strength and limitations

This study has a number of strengths and limitations.

It is important to note that for the children and families in this study, although there are no national guidelines for treatment of UHL, there are some guidelines about device candidacy (see the Supplementary Material). Thus, device choice for a child may not only depend on parental/child choice. In the United Kingdom, the National Health Service provides free hearing devices for all children at the point of service. Device type is not impacted by a plan under the health insurer of the family, and audiologists working for the National Health Service endeavour to provide an equal service for all children with hearing loss. There are no care guidelines for individuals with UHL; however, under current guidelines, the recommended care pathway for individuals with SSD in the National Health Service involves initially trialling a conventional hearing aid, followed by a CROS-aid, and then a BCD (see Supplementary Material for details). Bone-conduction implantations are funded by the National Health Service, but CIs for UHL/SSD are not. The current guidance for eligibility of individuals for consideration of a CI within the NHS includes a requirement for bilateral severe-profound hearing loss; children and adults with UHL/SSD in the United Kingdom following National Health Service care pathways are currently ineligible for this intervention.

A further limitation to this study is potential sampling bias since many children with mild losses can be missed; they may either not be picked up on UNHS or the parent may not follow up with audiological services following diagnosis.

Another limitation of the study is that we are not able to make inferences about device benefits since one cannot extrapolate from the reporting of wearing a particular device to the device being beneficial for the child in all environments.

A strength of this study is that there are advantages to following the same children over time in a single large centre, and the continuity of care for these children are reflected in consistently documented follow-up notes over a prolonged period. There was a consistency to record keeping, which contrasts to the data that are amalgamated from multiple sites and have different local policies and different record keeping regimens.

### Future work

We do not know why some children with UHL go on to require speech and language services and struggle to develop academically and behaviourally whilst others do not. The fitting of the hearing device, degree of hearing loss, and maternal education are key (42), but research is required investigating the contributing genetic and environmental factors.

Future research into how the brains processing of monaural cues are impacted by late identification of hearing loss or later aiding is needed, as these could impact the outcomes of the children (77). This is particularly important since some studies have shown that asymmetric hearing loss causes a reweighting of cues that are used and postulate that adapted monaural cues may be utilised for sound localisation (78, 79). A recent study in children with congenital conductive UHL showed that they may rely on monaural spectral cues for horizontal sound localisation (35). Context of listening cues can also be important for sound localisation and would be interesting to investigate (80).

Long-term follow-up into adulthood would be beneficial for this cohort to examine which and when are the best devices to trial for a child and the most favourable support services. It will also be important to understand the reasons the families do not engage with hearing services and to quantify the outcomes of their children. Setting up anti-bullying campaigns within schools could be vital for children with hearing loss as they are particularly vulnerable, and their device use is likely most useful in school. A core-outcome set of what is important for the children with UHL and their families is important to define.

It will be important to determine why particular children with UHL struggle in school, and whether instructional training for the parents and teachers improves the likelihood of a positive outcome for the child. This may be particularly important for the parents of the children with UHL who in a recent study underestimated the fatigue of their child (27).

Future research should concern the items that enable the children with UHL to succeed and discover biomarkers that can accurately quantify stress and the quality of life. These factors are likely to be complex, multi-faceted, and relate to their frustration, attention, anxiety, fatigue, peer relations, social confidence, independence in the classroom, and emotional maturity, which are the important variables in educational success for children. Researching these aspects of a child's education could be key to understanding their struggles and thus providing specific support they need to help them succeed.

### Conclusion

In our study, most children with UHL wore a BCD or a CROS-aid and reported they wore it for the duration of their school day. There was a very wide-ranging age of first device fit, but on average, it happened at school age (4.7 years). The children who trialled a BCD or hearing aid were fitted earlier at around 2.5 years. Additional support with speech and language via support services were available for three quarters of this cohort, but for those who were unable to access this support, it was primarily because the child did not meet the referral criteria. Several areas of provision of support services provided for the children with UHL are currently under resourced. Individualised treatment plans are essential for this distinct cohort but where devices are not trialled or worn then sound-field amplification systems in the nursery and school would improve all the outcomes of the children regardless of their hearing status.

The funding for genetic testing and consistent provision of support services, counselling, and anti-bullying campaigns within schools for this understudied group is vital.

## Data Availability

The raw data supporting the conclusions of this article will be made available by the authors, without undue reservation.
