# Supplementary material for: Characterisation of the treatment provided for children with unilateral hearing loss
Source: Front Pediatr. 2023 Jul 25;11:1197713. doi: 10.3389/fped.2023.1197713 (PMC10407268; doi:10.3389/fped.2023.1197713)
Supplement: Supplementary file 1 [file Datasheet1.pdf]

Supplementary Material:

Clinical commissioning policies for the NHS, UK

Commissioning Services for People with Hearing Loss: A framework for clinical commissioning groups (**https://www.england.nhs.uk/wp-content/uploads/2016/07/HLCF.pdf)**

Clinical Commissioning Policy: Bone conducting hearing implants (BCHIs) for hearing loss (all ages) - 2016

Implanted BCHDs are commissioned for use in adults and children as per manufacturers CE markings who have:

(1a) Unilateral or bilateral conductive or mixed hearing loss within the manufacturers fitting criteria; AND Stable bone conduction thresholds (≤15 dB deterioration in >2 frequencies in a 2 year period).

OR

(1b) Unilateral sensorineural hearing impairment (including SSD) where the better ear has bone-conduction hearing thresholds within the manufacturers fitting criteria including SSD;

AND (2) Trialled an ACHA or wireless CROS / BiCROS hearing aid for a minimum of 4 weeks, or who are anatomically or physiologically unable to undertake a trial of an ACHA;

AND (3) Trialled a BCHD on a softband or headband for a minimum of 14 days and show benefit in speech tests.

BCHDs will not be commissioned for:

- Patients with a bone disease that is unable to support an implant
- Patients who have a sensitivity or allergy to the materials used.
- Patients with physical, emotional or psychological disorders that, despite suitable treatment and support, would interfere with surgery or the ability to allow suitable rehabilitation such that significant benefit would be unlikely.

NHSP Clinical Group (2013) United Kingdom, NHS Guideline Guidelines for the Early Audiological Assessment and Management of Babies Referred NHSP, Ln 565 9.3 Mild bilateral loss & unilateral hearing loss The evidence base for the benefit of early intervention is for babies with bilateral permanent hearing loss of 40dBHL and greater, averaged over 0.5, 1, 2 & 4kHz. The current evidence does not support active early intervention or aiding for children with milder bilateral losses, or for unilateral hearing loss, and there are potential disbenefits as well as benefits (Carr et al 2012). However clinicians need to treat all such cases individually, and discuss and agree a plan with parents, including monitoring and review.
